# Supplementary material for: One-step induction of human GABAergic neurons promotes presynaptic development & synapse maturation
Source: bioRxiv. 2025 Nov 30:2025.06.30.662293. Preprint. [Version 3] doi: 10.1101/2025.06.30.662293 (PMC12265733; doi:10.1101/2025.06.30.662293)
Supplement: Supplement 2 [file NIHPP2025.06.30.662293v3-supplement-2.pdf]

bioRxiv preprint doi: https://doi.org/10.1101/2025.06.30.662293; this version posted November 30, 2025. The copyright holder for this preprint (which was not certified by peer review) is the author/funder, who has granted bioRxiv a license to display the preprint in perpetuity. It is made available under aCC-BY 4.0 International license.

Supplementary figures and table

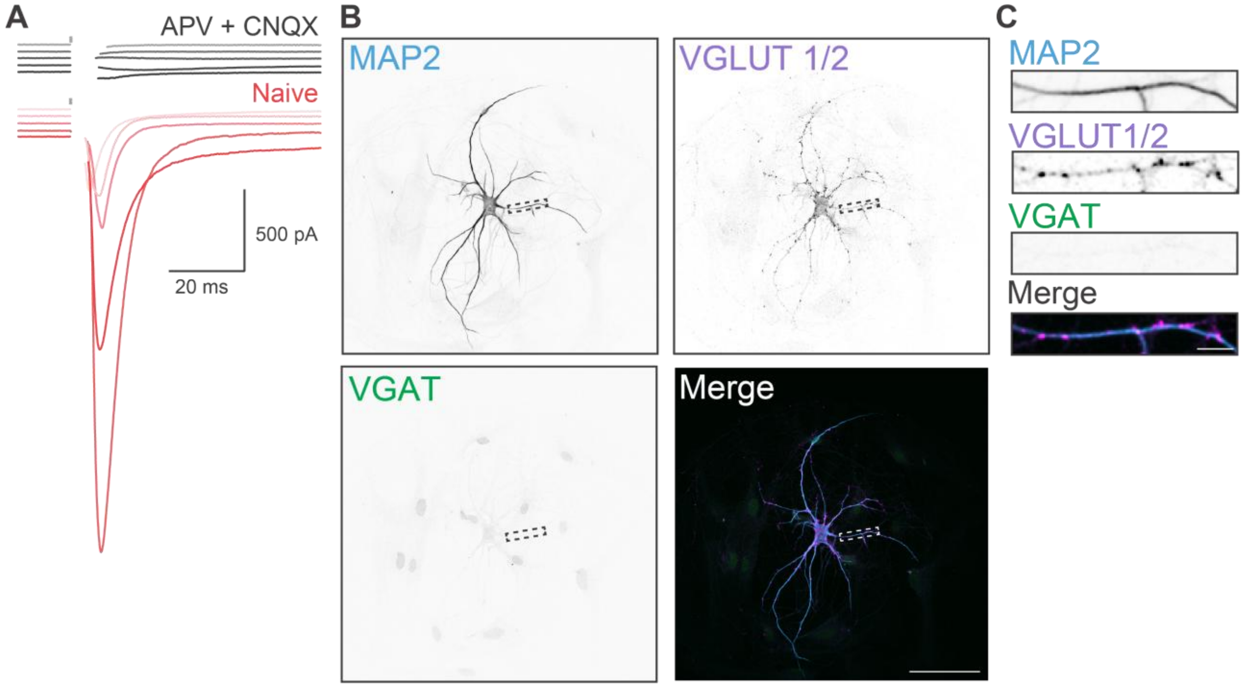

Figure S1. NGN2 neurons produce glutamatergic transmission

A Voltage clamp recordings of 6 WPI NGN2 autaptic neurons after stimulation, in normal ACSF (Naive) and in presence of glutamatergic receptor blockers (APV + CNQX). Synaptic transmission was fully abolished by blockers, illustrating that NGN2 neurons are glutamatergic. **B-C** Examples of immunocytochemistry for MAP2, VGAT and VGLUT1+2 performed on NGN2 autaptic neurons, with zoom in on a length of dendrite (**C**). Scale bar = 100 μm, zoom in scale bar = 10 μm

bioRxiv preprint doi: https://doi.org/10.1101/2025.06.30.662293; this version posted November 30, 2025. The copyright holder for this preprint (which was not certified by peer review) is the author/funder, who has granted bioRxiv a license to display the preprint in perpetuity. It is made available under aCC-BY 4.0 International license.

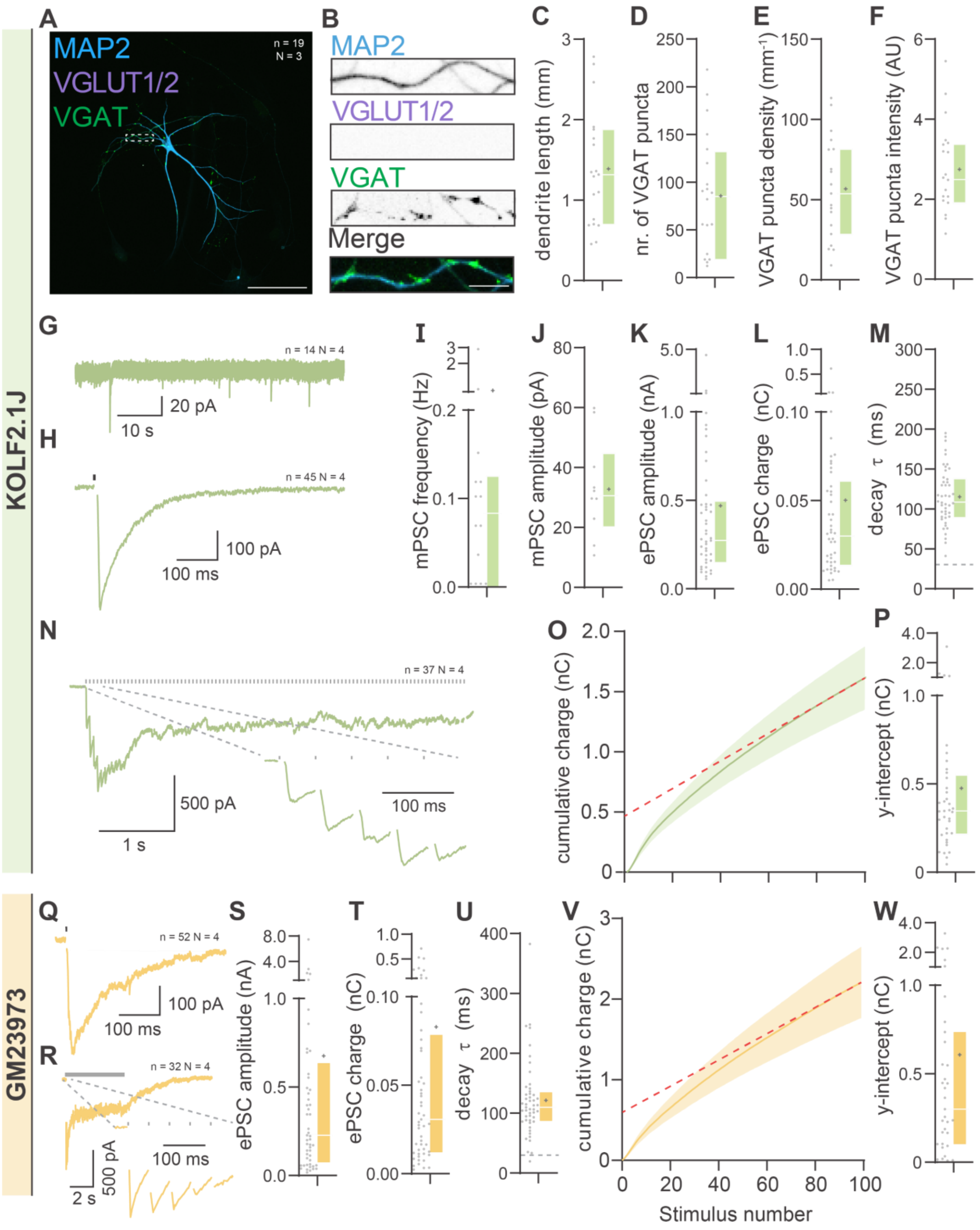

Figure S2. PiggyBac Ascl1/Dlx2 induction reliably generates GABAergic neurons in the KOLF2.1J and GM23973 line

A Representative images of PiggyBac (PB) Ascl1/Dlx2 (AD) neurons induced from KOLF1.2J iPSCs at 7 weeks post induction (WPI), stained for MAP2 as dendritic marker, and VGAT and VGLUT1/2 as GABAergic and glutamatergic markers, respectively. Scale bar: 100 μm **B** Zoom-

bioRxiv preprint doi: https://doi.org/10.1101/2025.06.30.662293; this version posted November 30, 2025. The copyright holder for this preprint (which was not certified by peer review) is the author/funder, who has granted bioRxiv a license to display the preprint in perpetuity. It is made available under aCC-BY 4.0 International license.

in on dendrite to illustrate punctate VGAT staining. Scale bar: 10 µm **C.** Total dendrite length and (**D**) number of GABAergic synapses per neuron. **E** Synaptic density and intensity (**F**). **G** Representative mPSC trace and (**H**) representative ePSC trace from PB-AD neurons induced from the KOLF2.1J line. **I** Frequency and (**J**) amplitude of miPSCs. **K** Amplitude, (**L**) charge and (**M**) decay time constant of iPSCs in PB-AD neurons generated from the KOLF2.1J line. **N** Representative trace of iPSCs evoked by 20Hz stimulation trains with zoom-in on first five pulses in KOLF2.1J. **O** Plot of cumulative iPSC amplitudes evoked by 20 Hz stimulus trains, with back-extrapolated linear fits displayed in red. **P** RRP estimate obtained from back-extrapolation of linear fits displayed in (**M**). **Q**. representative ePSC traces from PB-AD neurons induced from the GM23973 line. **R.** Representative trace of iPSCs evoked by 20Hz stimulation trains with zoom-in on first five pulses in GM23973. **S** Amplitude, (**T**) charge and (**U**) decay time constant of iPSCs in PB-AD neurons generated from the GM23973.1J line. **V** Plot of cumulative iPSC amplitudes evoked by 20 Hz stimulus trains, with back-extrapolated linear fits displayed in red. **W** RRP estimate obtained from back-extrapolation of linear fits displayed in (M). Boxplot whiskers extend the entire data range.

bioRxiv preprint doi: https://doi.org/10.1101/2025.06.30.662293; this version posted November 30, 2025. The copyright holder for this preprint (which was not certified by peer review) is the author/funder, who has granted bioRxiv a license to display the preprint in perpetuity. It is made available under aCC-BY 4.0 International license.

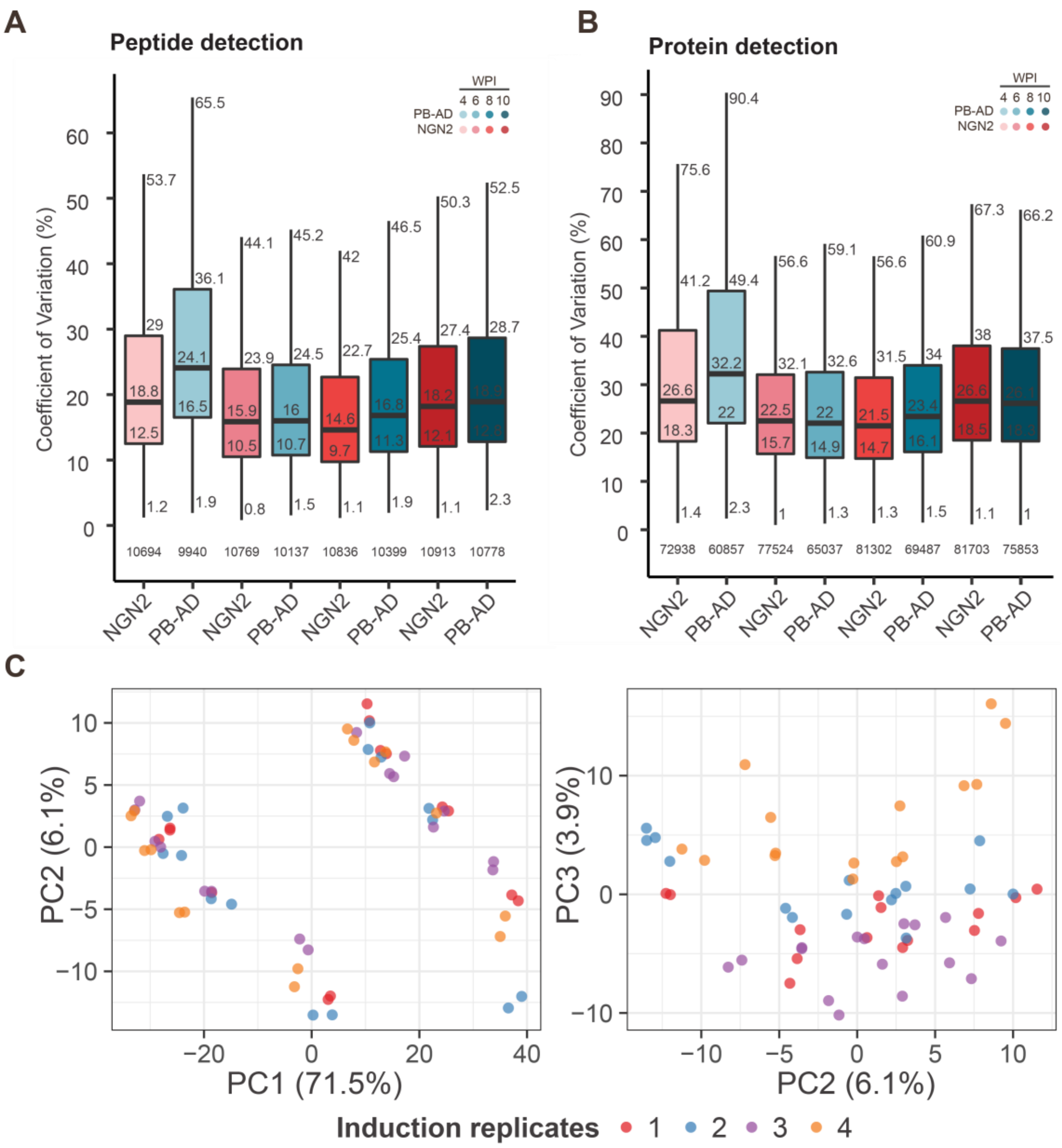

Figure S3. Peptide and protein detection count and variation is similar between cultures and timepoints.

A-B Boxplots showing variation of peptide (**A**) and protein (**B**) detection for all timepoints per culture, indicating median, 1st and 3rd quartiles, and minimum and maximum values. Number of detected peptides and proteins are indicated in the base of the graph. Median coefficient of variation for peptide and protein detection across groups ranged from 14.6% to 32.2%, reflecting consistent detection performance across replicates. **C** PCA plots showing potential batch effects due to induction. PC1 and PC2 show even spread of different inductions but PC3 (3.9%) shows slight separation based on induction and we therefore corrected for induction effect when performing differential abundance analysis.

bioRxiv preprint doi: https://doi.org/10.1101/2025.06.30.662293; this version posted November 30, 2025. The copyright holder for this preprint (which was not certified by peer review) is the author/funder, who has granted bioRxiv a license to display the preprint in perpetuity. It is made available under aCC-BY 4.0 International license.

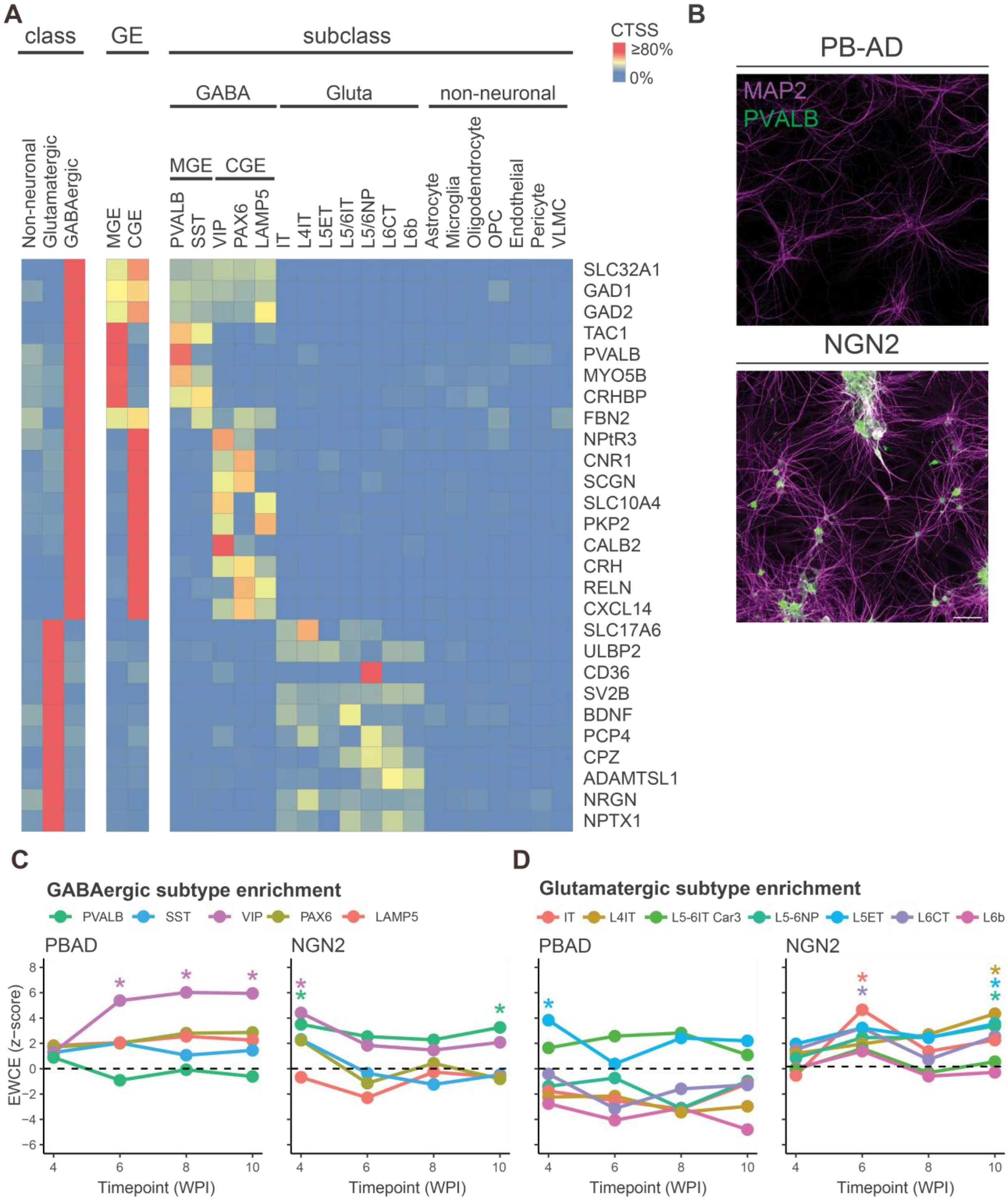

Figure S4. Cell type specificity scores for detected GABAergic proteins

A. Cell type specificity scores (CTSS) scores for detected GABAergic and glutamatergic proteins. CTSS are calculated with the expression weighted cell type enrichment tool.[41]

bioRxiv preprint doi: https://doi.org/10.1101/2025.06.30.662293; this version posted November 30, 2025. The copyright holder for this preprint (which was not certified by peer review) is the author/funder, who has granted bioRxiv a license to display the preprint in perpetuity. It is made available under aCC-BY 4.0 International license.

using single-cell RNA-sequencing data from Jorstad et al. [40] MGE and CGE refer to medial and caudal ganglionic eminences. **B.** Representative images of PB-AD and NGN2 neurons stained for MAP2 and PVALB. Scale bar = 100 µm. **C-D** Expression-weighted cell type enrichment (EWCE) analysis showing enrichment of proteins higher expressed in PB-AD or NGN2 for GABAergic **(C)** and glutamatergic subtypes **(D)**. PBAD-enriched proteins showed strongest enrichment for VIP^{+} interneurons at most timepoints, whereas NGN2-enriched proteins map at different timepoints to both GABAergic (PVALB^{+} and VIP^{+} interneurons) and glutamatergic subtypes. PB-AD-enriched proteins showed unexpected enrichment for L5ET neurons at 4 WPI, which reduced below significance threshold at later timepoints. IT and ET refer to intra- and extratelenchephalic-projecting neurons, and NP refers to near-projecting neurons – cell subclass annotations consistent with Jorstad et al. (2023).[40]

bioRxiv preprint doi: https://doi.org/10.1101/2025.06.30.662293; this version posted November 30, 2025. The copyright holder for this preprint (which was not certified by peer review) is the author/funder, who has granted bioRxiv a license to display the preprint in perpetuity. It is made available under aCC-BY 4.0 International license.

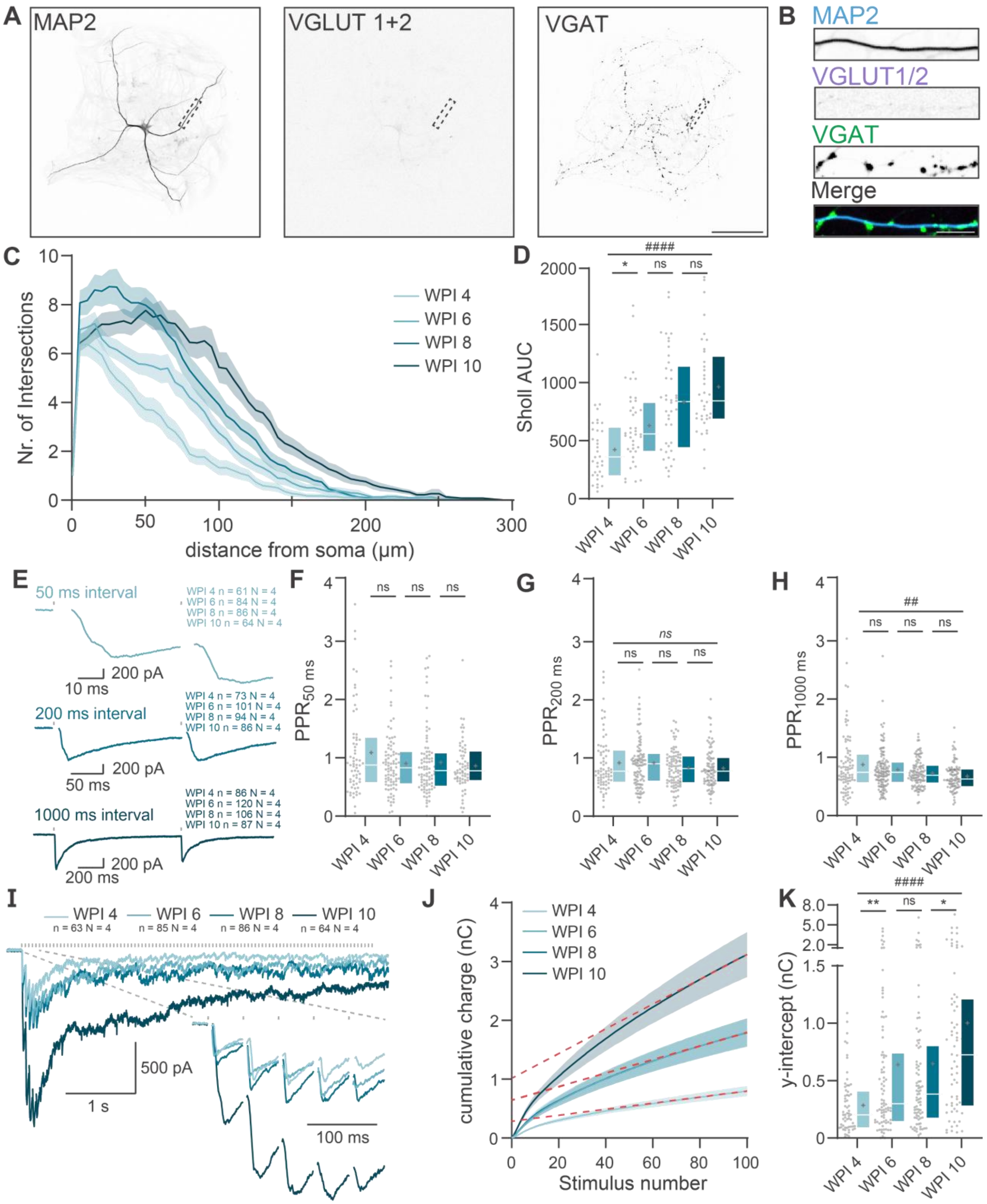

Figure S5. Extended morphological and functional development of PiggyBac Ascl1/Dlx2 induced neurons

A Representative images of an autaptic PiggyBac (PB) *Ascl1/Dlx2* (AD) induced neuron at week-post-induction 10, stained for MAP2 as dendritic marker, and VGAT and VGLUT1/2 as GABAergic and glutamatergic markers, respectively. Scale bar: 100 µm **B** Cut-out of dendrite

bioRxiv preprint doi: https://doi.org/10.1101/2025.06.30.662293; this version posted November 30, 2025. The copyright holder for this preprint (which was not certified by peer review) is the author/funder, who has granted bioRxiv a license to display the preprint in perpetuity. It is made available under aCC-BY 4.0 International license.

marked in A to illustrate individual synaptic puncta. Scale bar: 10 µm **C** Sholl analysis of PB-AD autaptic neurons at WPI 4, 6, 8 and 10. Shaded areas represent the SEM. **D** Quantification of the area under the curve (AUC) of the Sholl histogram shown in (**C**) **E** Representative traces of paired-pulse ePSCs at a 50, 200 and 1000 ms interval at 10 WPI. **F** Quantified paired-pulse amplitude ratios (PPR) at a 50 ms, 200 ms (**G**) and 100 ms (**H**) interval at 4, 6, 8 and 10 WPI. **I** Representative traces of iPSCs evoked by 20Hz stimulus trains from PB-AD neurons at 4, 6, 8 and 10 WPI with zoom in on first five pulses. **J** Plot of cumulative iPSC charge evoked by 20 Hz stimulus trains, with back-extrapolated linear fits displayed in red. Note, WPI 4 and WPI 6 data overlap. **K** RRP estimate obtained from back-extrapolation of linear fits displayed in (**J**).

bioRxiv preprint doi: https://doi.org/10.1101/2025.06.30.662293; this version posted November 30, 2025. The copyright holder for this preprint (which was not certified by peer review) is the author/funder, who has granted bioRxiv a license to display the preprint in perpetuity. It is made available under aCC-BY 4.0 International license.

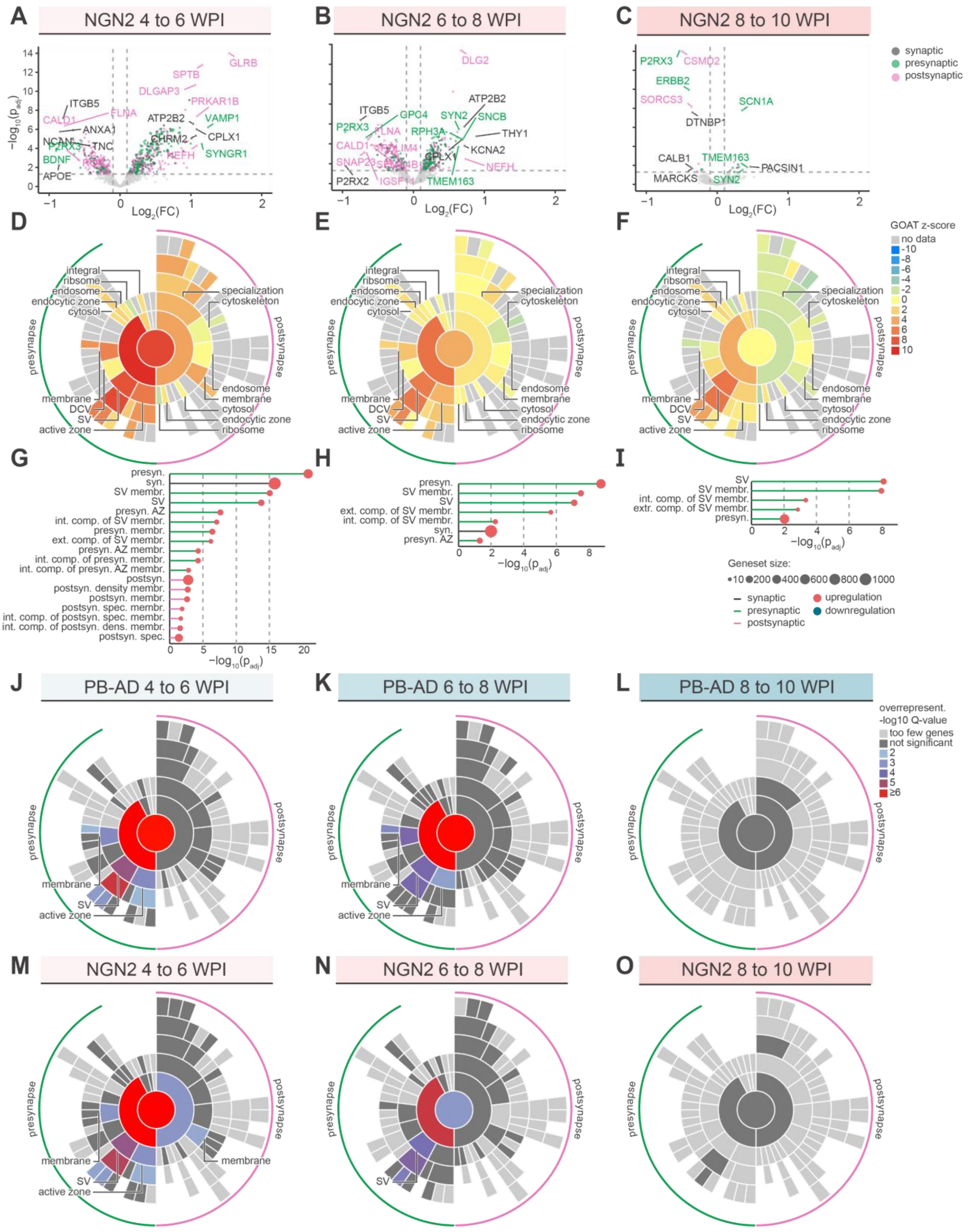

bioRxiv preprint doi: https://doi.org/10.1101/2025.06.30.662293; this version posted November 30, 2025. The copyright holder for this preprint (which was not certified by peer review) is the author/funder, who has granted bioRxiv a license to display the preprint in perpetuity. It is made available under aCC-BY 4.0 International license.

*Figure S***6**. *Differential expression of synaptic genes in NGN2 and PB-AD neurons*

A-C. Volcano plots showing regulation of synaptic proteins in NGN2 cultures between 6 and 4 (**A**), 8 and 6 (**B**), and 10 and 8 WPI (**C**). Only SYNGO-annotated proteins are plotted and colour coding refer to significantly regulated synaptic (black) exclusively presynaptic (green) or postsynaptic (pink) annotations. Proteins were regarded significantly regulated when adjusted p-value < 0.05 (dashed horizontal line) and $\log_2$ (FC) > 0.1 or < -0.1 (dashed vertical lines). **D-F**. SYNGO plots showing enrichment scores (z-scores) derived from gene-set enrichment analysis with GOAT using SYNGO cellular compartment gene sets for 4 to 6 (**D**), 6 to 8 (**E**) and 8 to 10 WPI (**F**). Negative z-scores (blue) reflect enrichments primarily driven by downregulated proteins and vice versa for positive z-scores (red). **G-I.** Lollipop charts showing significant SYNGO terms with their corresponding adjusted p-values and the number of genes part of the gene set. **J-O.** SYNGO overrepresentation analysis of upregulated proteins in PB-AD (**J-L**) and NGN2 (**M-O**) per timepoint. Foreground are significantly upregulated proteins and background are all tested genes in corresponding contrast.

bioRxiv preprint doi: https://doi.org/10.1101/2025.06.30.662293; this version posted November 30, 2025. The copyright holder for this preprint (which was not certified by peer review) is the author/funder, who has granted bioRxiv a license to display the preprint in perpetuity. It is made available under aCC-BY 4.0 International license.

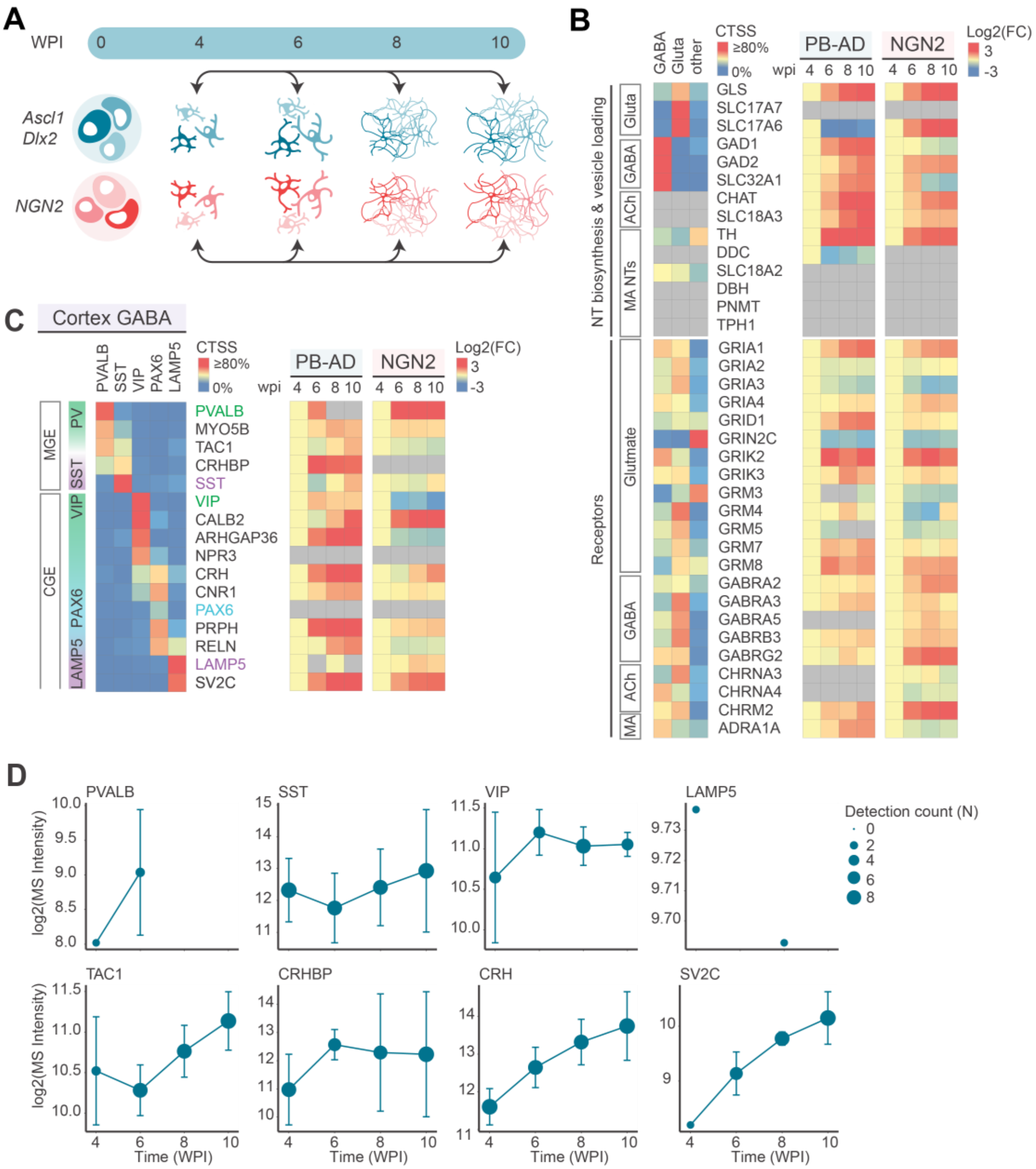

Figure S7. PiggyBac Ascl1/Dlx2 and NGN2 neurons express markers of diverse GABAergic neuron subtypes

A. Contrasts used to determine cell type specification. **B** and **C** Heatmaps that show temporal expression profiles relative to 4 WPI (log_2 (FC)) and cell type specificity scores (CTSS). **B** Typical proteins required for neurotransmitter (NT) synthesis, vesicle loading, and receptors. Undetected proteins are visualized for NT biosynthesis and vesicle loading but not for receptors. **C** Canonical GABAergic subtype markers and proteins with high cell type specificity for GABAergic subtypes and for medial or caudal ganglionic eminences (MGE or CGE). **D.** Line plots show sum expression of canonical GABAergic subtype markers (top) and other proteins with GABAergic cell type specificity. Error bars represent SD and dot size indicate detection count.

bioRxiv preprint doi: https://doi.org/10.1101/2025.06.30.662293; this version posted November 30, 2025. The copyright holder for this preprint (which was not certified by peer review) is the author/funder, who has granted bioRxiv a license to display the preprint in perpetuity. It is made available under aCC-BY 4.0 International license.

Supplemental table 1: SYNGO Proteins with significant expression changes from 8 to 10 WPI in PB-AD neurons

Protein	new at WPI 10	log_2fc	FDR-adjusted p-value
CNTNAP4	yes	1.05	1.50E-26
SLITRK1	yes	0.97	7.75E-23
CDH1	yes	0.91	4.81E-20
EPHA7	yes	0.90	1.26E-19
ITGB3	no	0.80	1.72E-15
PRRT1	yes	0.74	1.35E-13
NPTX2	no	0.73	4.68E-13
ITGA2	no	0.67	3.46E-11
SLC17A5	yes	0.67	5.09E-11
KCNQ2	yes	0.65	1.77E-10
ELFN1	no	0.65	2.09E-10
CYTH1	no	0.65	2.21E-10
TNC	no	0.64	3.74E-10
NRGN	yes	0.63	6.71E-10
NGDN	yes	0.63	6.73E-10
GPR158	no	0.62	1.43E-09
RELN	yes	0.57	3.53E-08
SCG2	no	0.78	2.18E-03
TMEM163	no	0.62	7.64E-03
PTPRN	no	0.59	2.24E-02
NEFH	no	0.40	2.24E-02
IGSF8	no	0.40	2.24E-02
CNTNAP1	no	0.34	3.78E-02
ATP2B4	no	0.30	3.78E-02
